# Manganese Luminescent Centers of Different Valence in Yttrium Aluminum Borate Crystals

**DOI:** 10.3390/ma16020537

**Published:** 2023-01-05

**Authors:** Anastasiia Molchanova, Kirill Boldyrev, Nikolai Kuzmin, Alexey Veligzhanin, Kirill Khaydukov, Evgeniy Khaydukov, Oleg Kondratev, Irina Gudim, Elizaveta Mikliaeva, Marina Popova

**Affiliations:** 1 Institute of Spectroscopy, Russian Academy of Sciences, Troitsk, 108840 Moscow, Russia; 2 Landau Phystech School of Physics and Research, Moscow Institute of Physics and Technology, 141701 Dolgoprudny, Russia; 3 Faculty of Geology, Lomonosov Moscow State University, 119991 Moscow, Russia; 4 National Research Center “Kurchatov Institute”, 123182 Moscow, Russia; 5 Federal Scientific Research Center “Crystallography and Photonics”, Russian Academy of Sciences, 119333 Moscow, Russia; 6 Kirensky Institute of Physics, Siberian Branch of the Russian Academy of Sciences, Akademgorodok, 660036 Krasnoyarsk, Russia; 7 Branch “Aprelevka Department of VNIGNI”, Federal State Budgetary Institution “All-Russian Research Geological Oil Institute”, 143360 Aprelevka, Russia

**Keywords:** manganese, YAl_3_(BO_3_)_4_:Mn crystal, XANES spectroscopy, high-resolution optical spectroscopy, photoluminescence

## Abstract

We present an extensive study of the luminescence characteristics of Mn impurity ions in a YAl_3_(BO_3_)_4_:Mn crystal, in combination with X-ray fluorescence analysis and determination of the valence state of Mn by XANES (X-ray absorption near-edge structure) spectroscopy. The valences of manganese Mn^2+^(d^5^) and Mn^3+^(d^4^) were determined by the XANES and high-resolution optical spectroscopy methods shown to be complementary. We observe the *R*_1_ and *R*_2_ luminescence and absorption lines characteristic of the ^2^*E ↔* ^4^*A*_2_ transitions in d^3^ ions (such as Mn^4+^ and Cr^3+^) and show that they arise due to uncontrolled admixture of Cr^3+^ ions. A broad luminescent band in the green part of the spectrum is attributed to transitions in Mn^2+^. Narrow zero-phonon infrared luminescence lines near 1060 nm (9400 cm^−1^) and 760 nm (13,160 cm^−1^) are associated with spin-forbidden transitions in Mn^3+^: ^1^*T*_2_ → ^3^*T*_1_ (between excited triplets) and ^1^*T*_2_ → ^5^*E* (to the ground state). Spin-allowed ^5^*T*_2_ → ^5^*E* Mn^3+^ transitions show up as a broad band in the orange region of the spectrum. Using the data of optical spectroscopy and Tanabe–Sugano diagrams we estimated the crystal-field parameter *Dq* and Racah parameter *B* for Mn^3+^ in YAB:Mn as *Dq* = 1785 cm^−1^ and *B* = 800 cm^−1^. Our work can serve as a basis for further study of YAB:Mn for the purposes of luminescent thermometry, as well as other applications.

## 1. Introduction

**Figure 1:**
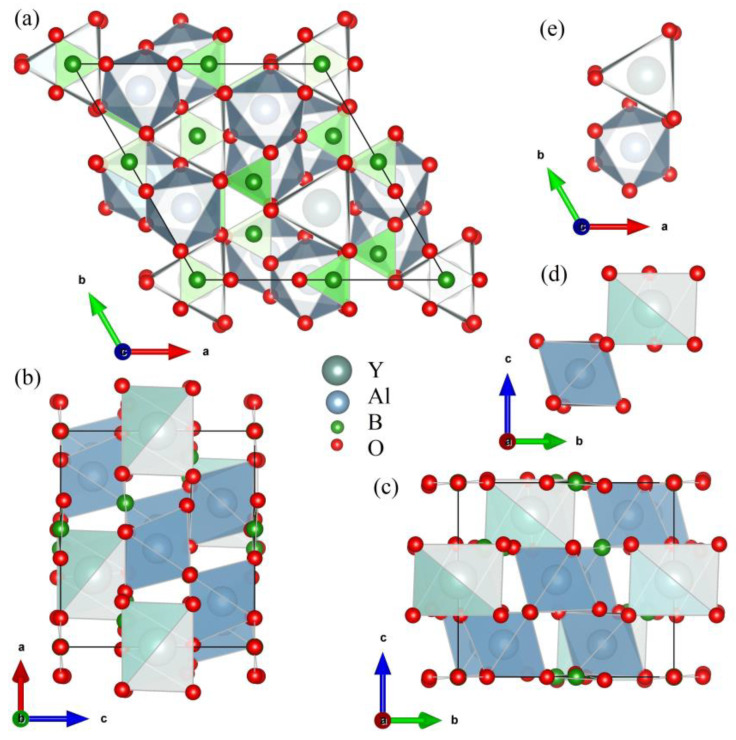
Projections of the YAl_3_(BO_3_)_4_ unit cell along the *c* axis (**a**), the *b* axis (**b**), and the *a* axis (**c**). Projections of the YO_6_ trigonal prism and AlO_6_ distorted octahedron in the YAl_3_(BO_3_)_4_ unit cell, along *a* axis (**d**) and the *c* axis (**e**).

Crystals of yttrium-aluminum borate YAl_3_(BO_3_)_4_ (YAB) have the structure of the mineral huntite CaMg_3_(CO_3_)_4_ with the non-centrosymmetric space group *R*32 of the trigonal system [1]. Figure 1 shows different projections of the YAB unit cell. The crystal structure is formed by layers that are perpendicular to the crystallographic *c* axis and consist of distorted YO_6_ prisms, AlO_6_ octahedra, and BO_3_ groups of two types (B1O_3_ and B2O_3_). Y^3+^ ions in YO_6_ prisms are surrounded by six oxygen atoms of one type and occupy sites with the *D*_3_ point symmetry group. The point group of AlO_6_ octahedra is *C*_2_. AlO_6_ octahedra linked together by their edges form spiral chains running along the *c* axis. The Y^3+^ ions are situated between three such chains and link the chains together. YO_6_ prisms are isolated from each other, having no oxygen atoms in common, which, in the case of a substitution of the Y^3+^ ions by rare-earth or transition metal ions, results in low luminescence quenching [2]. This property, together with high optical nonlinearity and excellent physical characteristics and chemical stability, make YAB extremely interesting for many applications. Doped with various rare-earth and transition metal ions, YAB crystals are well-known phosphors, promising for use as materials for display panels, lasers, scintillators, LEDs, luminescent thermometers, and in medical imaging [3,4,5,6,7,8,9,10,11,12,13,14,15,16,17,18]. YAB crystals doped with Nd^3+^ [8,13], Yb^3+^ [11,12,14], Er^3+^/Yb^3+^ [10], and Yb^3+^/Tm^3+^ [16] are well-known media for self-frequency doubling, self-frequency summing, and up-conversion lasers. Tunable anti-Stokes ultraviolet–blue light generation was demonstrated using a random laser based on Nd_0.10_Y_0.90_Al_3_(BO_3_)_4_ [3]. YAB:Eu^3+^/Tb^3+^ phosphors were proposed for eye-friendly white LEDs [6]. In addition, YAB:Cr is being investigated as a material for LEDs [17]—in particular, as a phosphor for plant growth LEDs—with excellent thermal stability and high luminescent yield [5]. Recently, impressive applications of YAB:Pr^3+^/Gd^3+^ and YAB:Cr^3+^ in luminescent thermometry were reported [4,15]. In Ref. [15], it was proposed to use several excited levels of the Gd^3+^ ion in YAB doped with Pr^3+^ and Gd^3+^ ions in the UV region of the spectrum to implement a Boltzmann thermometer operating from 30 to 800 K. The UV region allowed detuning from background thermal radiation even at the highest temperatures. In this case, excitation was carried out at a wavelength of 450 nm using an inexpensive commercial LED into the absorption band of the Pr^3+^ ion, followed by the up-conversion energy transfer Pr^3+^ → Gd^3+^. In Ref. [4], a combination of optical heating and luminescent thermometry in YAB:Cr^3+^ was realized. Here, the temperature-dependent ratio of emission intensities for the ^4^*T*_2_→ ^4^*A*_2_ and ^2^*E*→ ^4^*A*_2_ transitions of Cr^3+^ was used to measure the temperature.

We note that the Mn^4+^ ion has the same valence electron shell as the Cr^3+^ ion (d^3^) and is also used for luminescent thermometry [19]. Compounds with Mn^3+^(d^4^) exhibit broadband, extremely temperature-sensitive luminescence in the near-IR and visible spectral ranges [20,21], due to which compounds with Mn^3+^ are also topical materials for thermoluminescent sensors. Cryogenic luminescence ratiometric thermometry based on the diverse thermal quenching behaviors of Mn^3+^ and Mn^4+^ in manganese-doped garnet-type Ca_3_Ga_2_Ge_3_O_12_ single crystals was explored [22]. Tb^3+^ and Mn^3+^ co-doped La_2_Zr_2_O_7_ nanoparticles were recently suggested as a promising material for dual-activator ratiometric optical thermometry [23]. Mn^2+^(d^5^)-containing phosphors exhibit bright broadband luminescence with a maximum from the red to green region of the spectrum, depending on the particular matrix [24,25,26]. In light of all of the above, it is of interest to study the luminescent properties of YAB doped with manganese.

We are aware of only one work on YAB:Mn spectroscopy ([27]). Only the room-temperature spectra were measured in Ref. [27]. Three lines characteristic of ^2^*E* → ^4^*A*_2_ emission of ions with a d^3^ electronic configuration were detected in the YAB:Mn room-temperature luminescence spectra [27]. The authors assigned these lines to Mn^4+^(d^3^). The results of electron paramagnetic resonance (EPR) showed that Mn introduced into YAB at low concentrations predominantly occupied the yttrium-ion sites in the crystal structure, its valence in this case being 2+ [28]. Two broad bands peaked at 544 and 637 nm were observed in the room-temperature luminescence spectrum of YAB:Mn and assigned to the transition from the ^4^*T*_1_ state of the Mn^2+^ ion, split by a low-symmetry component of the crystal field, to the ground state ^6^*A*_1_ [27]. Since the Mn^2+^ and Mn^4+^ ions presumably replace the trivalent Y^3+^ and Al^3+^ cations, respectively, the question of charge-compensation arises. The formation of charge-compensating Mn^2+^–Mn^4+^ dimers was suggested in [27]. In this work, we continue the study of the valence states of manganese in YAB:Mn using XANES spectroscopy and high-resolution broadband temperature-dependent optical spectroscopy, and obtain extensive data on the luminescence of Mn impurity centers of various valences in YAl_3_(BO_3_)_4_.

## 2. Materials and Methods

YAl_3_(BO_3_)_4_:Mn crystals were obtained by the flux method of crystal growth in the laboratory of L.N. Bezmaternykh at the Kirensky Institute of Physics of the Siberian Branch of the Russian Academy of Sciences in Krasnoyarsk. They were grown on seeds in platinum crucibles with a volume of 50 mL. The composition of the system during the flux crystal growth was 85 wt.% (Bi_2_Mo_3_O_12_ + 2B_2_O_3_ + 0.5Li_2_MoO_4_) + 15 wt.% YAl_3_(BO_3_)_4_ with the addition of Mn_2_O_3_. The temperature regime consisted of heating the solution-melt to 1100 °C and then slowly cooling at a rate of 0.5 °C/h for 48 h. Note that manganese oxide Mn_2_O_3_ decomposes in air at temperatures above 800 °C to form Mn_3_O_4_ (Mn^2+^Mn^3+^_2_O_4_) [29]. High-purity reagents were used in flux crystal growth. Cr (0.001%) and Pb (0.0005%) impurities in Al_2_O_3_ as well as Nd_2_O_3_ and Sm_2_O_3_ (<0.0001%) in Y_2_O_3_ have been reported on certificates and are of interest for further discussion.

Powder X-ray diffraction on the grown crystals at room temperature was performed on a Thermo Fisher Scientific ARL X’tra diffractometer (Basel, Switzerland) equipped with a Dectris MYTHEN2 R 1D detector (Cu K_α1,2_ radiation). The operational voltage and current were 40 kV and 40 mA, respectively. Powder diffraction patterns were obtained in continuous mode at a rate of 2°/min in Bragg–Brentano geometry over an angle range of 10° ≤ 2*θ* ≤ 90°. The unit cell parameters of YAl_3_(BO_3_)_4_:Mn were refined by the Le Bail method using the JANA2006 program [30]. All parameters were refined by the least-squares method. The pseudo-Voigt function was used as the peak profile function. The structural data for YAl_3_(BO_3_)_4_ (sp. gr. *R*32, *a* = 9.295(3) Å, *c* = 7.243(2) Å, α = β = 90°, γ = 120°) were used as the initial structural parameters [31].

X-ray fluorescence analysis was carried out on a Bruker M4 Tornado analyzer. Absorption and luminescence spectra in the near-IR and visible ranges (5000–16,000 cm^−1^) with a spectral resolution up to 0.2 cm^−1^ were recorded on a spectrometer Bruker IFS 125HR (Bruker Optik GmbH, Ettlingen, Germany). Luminescence spectra in the visible and UV ranges (9000–20,500 cm^−1^) with a spectral resolution up to 3 cm^−1^ were registered using a OceanInside HDX spectrometer. The sample was cooled down to 5 K using a Cryomech ST403 closed-cycle helium cryostat (Syracuse, NY, USA). X-ray absorption spectra near the manganese K-edge were measured at the “Structural Materials Science” beamline at the Kurchatov Synchrotron Radiation Source [32] by X-ray fluorescence yield. Luminescence excitation spectra were recorded at a liquid nitrogen temperature (77 K) on a Fluorolog^®^-3 spectrofluorometer at the Institute of Photonic Technologies of the Federal Research Center “Crystallography and Photonics” of the Russian Academy of Sciences.

## 3. Results and Discussion

### 3.1. X-ray Diffraction (XRD) Analysis

**Figure 2:**
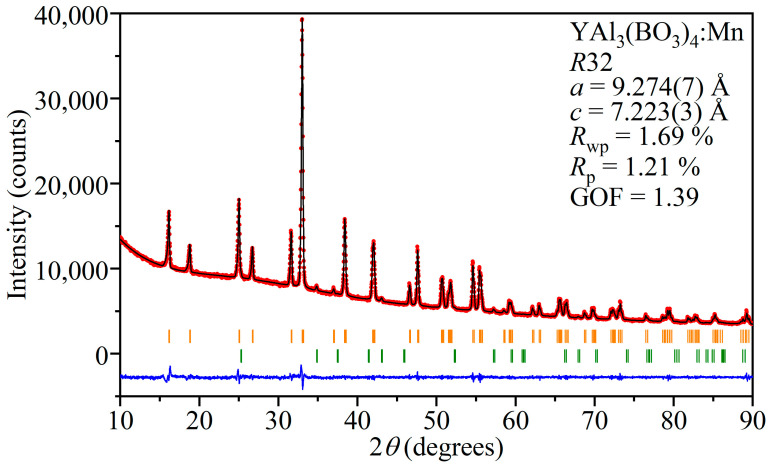
Final convergence of the Le Bail refinement for YAl_3_(BO_3_)_4_:Mn. The experimental diffraction pattern is shown by red circles (I*_obs_*); the black line (I*_calc_*) is the calculated diffraction pattern and the residual intensities (I*_obs_*-I*_calc_*) are shown as the blue line. The orange bars indicate the YAl_3_(BO_3_)_4_ reflections, and the green bars indicate the Al_2_O_3_ reflections.

XRD was used for the fingerprint characterization and investigation of the structural phases in the crystalline state. XRD patterns were analyzed by the Le Bail method in order to extract the parameters of the unit cell. The refined unit cell parameters were *a* = 9.274(7) Å, *c* = 7.223(3) Å, α = β = 90°, and γ = 120°. The convergence of the Le Bail approximation is shown in Figure 2. It can be seen that the diffraction pattern is well described, as indicated by the low *R*-factor values and small difference between the calculated and experimental diffraction patterns. The figure shows additional reflections of the Al_2_O_3_ phase. Their presence is explained by the fact that a corundum mortar was used in the preparation of the powder samples.

### 3.2. X-ray Fluorescence Analysis

The concentration of manganese ions was determined by X-ray fluorescence analysis to be 0.87 at.%. In addition, the presence of 1.18 at.% Bi was found, which is explained by its presence in the composition of the solvent. Insignificant amounts of potassium, calcium, titanium, and iron impurities were also found (see Table 1).

### 3.3. X-ray Absorption Spectroscopy

**Figure 3:**
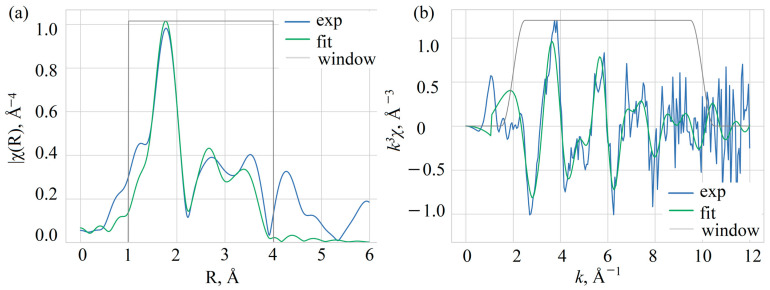
Fourier transform (**a**) and the oscillating part (**b**) of the EXAFS spectrum of Mn in YAB:Mn.

To address Mn ion oxidation state and position in the crystal structure, the fine structure of the X-ray absorption spectrum at the K-edge of manganese was measured. The EXAFS (Extended X-ray Absorption Fine Structure) spectrum was processed and analyzed using the software package IFEFFIT, version 1.2.11c [33,34]. The measured XAFS data were first processed by the ATHENA program of this package to merge four independently measured spectra, normalize the spectrum to a unity-height jump, and obtain the oscillating part of the spectrum. The fine structure of the X-ray absorption spectrum obtained in this manner after the *K*-jump was then used for the structural analysis (Figure 3). The local structure of manganese ions in the crystal was analyzed by fitting the EXAFS spectra at the K-edge of Mn to the model of the local structure based on the crystal structure of YAB [31]. Two distinct models were used for the fitting. The first model includes a manganese atom in the yttrium position. The second model takes into account the partial occupation of aluminum positions by manganese atoms. Since the positions of yttrium and aluminum differ significantly in metal–oxygen distances in the first coordination sphere—2.3 and 2.0 Å, respectively—this was taken into account by introducing an additional Mn-O scattering path of shorter length. To estimate the occupancy of the aluminum position, the coordination numbers for the two nearest oxygen coordination spheres were chosen so that their sum was fixed equal to six (Table 2). The distance for this shorter path was set to 2.052 Å to obtain a stable fit. Other parameters determined by fitting the EXAFS spectra are the distances between the absorbing and neighboring atoms *R_j_* and the Debye–Waller factors *σ_j_^2^* common to atoms of the same type. The errors for the Debye–Waller factors are quite large, since we can only use the spectrum up to *k* = 10 Å^−1^ due to the relatively high noise levels at large *k*. This leads to a significant correlation of the Debye–Waller factors with the overall amplitude of the EXAFS oscillations and to high uncertainty values. The refinement also included the Fermi energy shift Δ*E*_0_ and the attenuation coefficient of the signal amplitude *S*_0_^2^. The fitting ranges in *k* space and in *R* space were 2–10 Å^−1^ and 1–4 Å, respectively. The quality of the fit is characterized by the factor *R*_f_, which indicates the percentage mismatch between the data and the model.

Table 2 shows that the two models do not differ in *R*_f_, i.e., manganese in aluminum positions does not contribute much to the EXAFS signal. Thus, one can conclude that the occupation of aluminum sites by manganese atoms is rather small. To estimate this occupation, the coordination numbers for the split coordination sphere of oxygen can be used. For a shorter distance, it was determined to be 0.7, so occupancy can be estimated as no more than 10%. It should be noted that this estimate shows the sensitivity of the EXAFS method for this quantity, since the error bars are also of the same order. The distances determined by the EXAFS fit correspond to the local structure of the yttrium site. The distance to oxygen in the first coordination sphere was determined to be 2.26 ± 0.03 Å, which is slightly smaller than the Y-O distance in the YAB structure (2.313 Å) [31].

**Figure 4:**
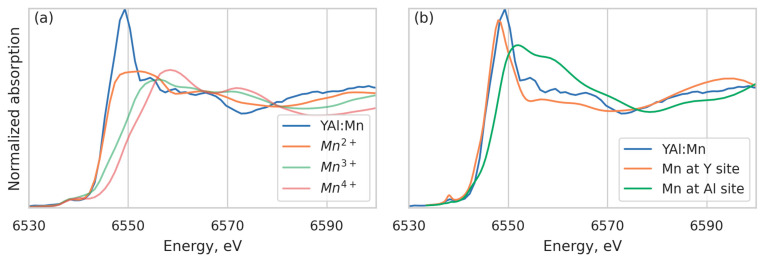
K-edge absorption spectra of Mn in YAB:Mn compared with the “reference” Mn^2+^, Mn^3+^, and Mn^4+^ spectra (**a**); calculated XANES spectra for Mn at the Y^3+^ and Al^3+^ sites in YAB crystal structure compared with experimental spectrum (**b**).

The XANES part of the spectrum also provides valuable information. The position of the K-edge can be used to obtain the oxidation state of Mn [35]. Comparing the spectrum with manganese references Mn(BO_2_)_2_, Mn_2_O_3_, and MnO_2_ with the oxidation states Mn^2+^, Mn^3+^, and Mn^4+^, respectively, measured on the same beamline, we can see that the edge position coincides with the Mn^2+^ reference (Figure 4a), which means that most manganese atoms are in the Mn^2+^ state. We cannot decompose the spectrum into a linear combination of references, since they are irrelevant to the local structure of the YAB specimen; the admixture of manganese in higher oxidation states can be roughly estimated as 10%. In addition, we calculated the XANES spectrum using the FDMNES code [36] with two structural models, corresponding to the manganese atom at the Y and Al sites in the YAB crystal structure, respectively (Figure 4b). The experimental data are reproduced only for Mn at the Y position, which confirms the conclusions of the EXAFS data analysis and is consistent with the EPR data [28]. From this point of view, a smaller Y-O distance than in YAB can be explained by a smaller ionic radius of Mn^2+^ (0.83 Å) as compared to Y^3+^ (0.90 Å) [37].

### 3.4. Optical Spectroscopy

**Figure 5:**
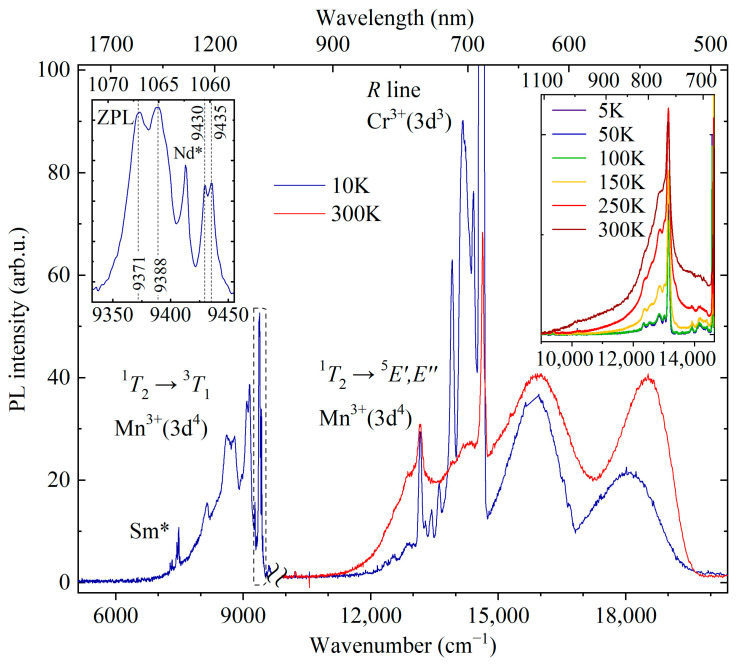
PL spectrum of YAB:Mn in a broad spectral range. The break separates the parts of the spectrum recorded on the Bruker IFS HR125 spectrometer (5100–9900 cm^−1^ at 10 K, λ_ex_ = 450 nm) and on the OceanInside HDX spectrometer (9900–20,500 cm^−1^ at 10 and 300 K, λ_ex_ = 488 nm). The left inset shows the region of zero-phonon lines in an enlarged scale. The right inset presents the region around the line 13,160 cm^−1^ at different temperatures (5–300 K, Bruker IFS HR125 spectrometer). The lines due to uncontrolled Sm^3+^ and Nd^3+^ impurities are marked as Sm* and Nd*, respectively.

Figure 5 shows the photoluminescence (PL) spectrum of YAB:Mn in a broad spectral range. The near-IR luminescence was recorded with the Bruker 125 HR Fourier spectrometer, while for the visible part of the PL spectrum an OceanInside HDX spectrometer was used. Relative intensities of these two parts cannot be compared.

**Figure 6:**
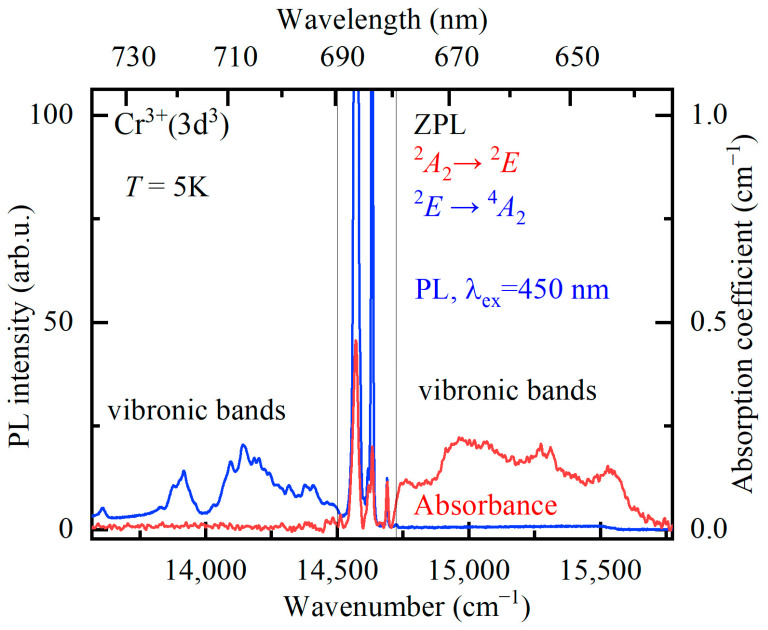
Absorption (red curve) and luminescence (blue curve, excitation wavelength λ_ex_ = 450 nm) spectra of YAB:Mn at the temperature *T* = 5 K in the region (indicated by gray thin vertical lines) of zero-phonon *R* lines and the region of associated vibronic bands.

**Figure 7:**
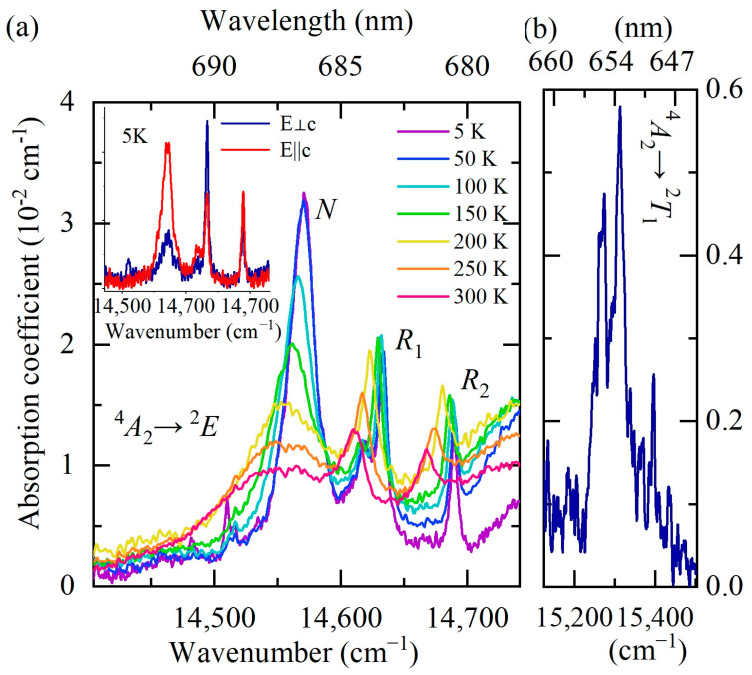
Unpolarized absorption spectra of YAB:Mn at different temperatures in the spectral range of zero-phonon *R* lines. The inset shows the spectra at *T* = 5 K for two polarization directions of the incident light: E||*c* (red trace) and E⊥*c* (blue trace) (**a**). Absorption spectrum at *T* = 5 K in the region of the ^4^*A*_2_ → ^2^*T*_1_ transition (**b**).

**Figure 8:**
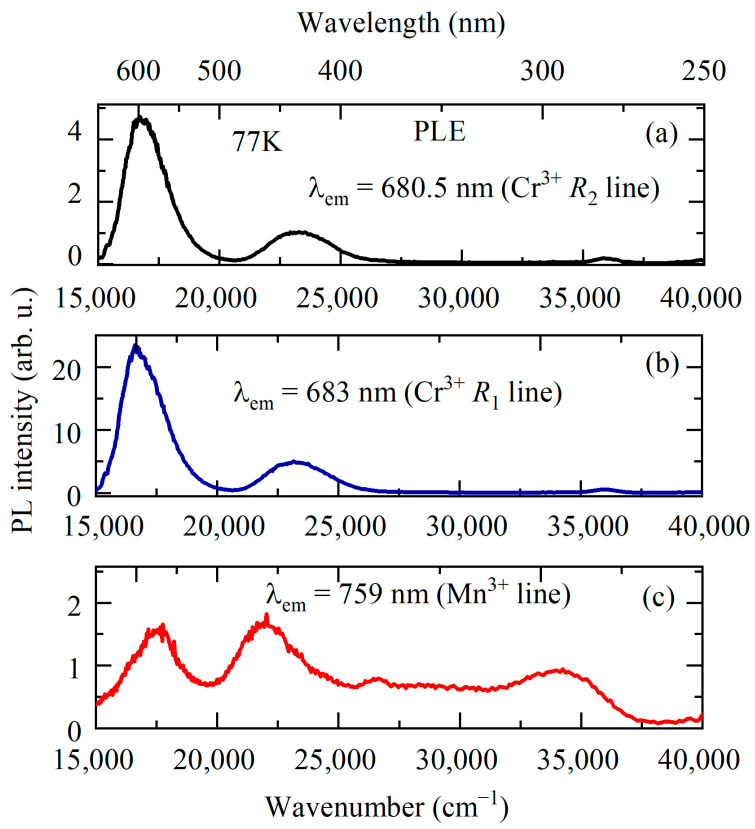
PL excitation spectra for YAB:Mn at *T* = 77 K monitored at 680.5 (**a**), 683 (**b**), and 759 (**c**) nm.

A strong relatively narrow peak at about 685 nm is observed in the room-temperature low-resolution PL spectrum. Previously, three narrow peaks with maxima at 682, 684, and 686 nm were reported in the room-temperature luminescence spectrum of YAB:Mn, and two of them were attributed to the *R*_1_ and *R*_2_ lines of Mn^4+^ [27]. The Mn^4+^ ion has the same valence electron shell structure as Cr^3+^(d^3^). Narrow *R* lines in the spectra of d^3^ ions arise due to spin-forbidden transitions from the excited orbital doublet ^2^*E* to the ground orbital singlet ^4^*A*_2_. In a low-symmetry crystal field, the ^2^*E* level, which is doubly degenerate in the cubic crystal field approximation, splits into two components, so that the *R*_1_ and *R*_2_ lines can be observed. We were able to observe peaks at the same wavelengths as in [27], both in the luminescence and absorption room-temperature spectra. However, a more detailed study of the temperature-dependent absorption, PL, and PL excitation spectra led us to the conclusion that those are *R* lines of uncontrolled Cr^3+^ impurity. Figure 6, Figure 7 and Figure 8 display these spectra.

Figure 6 shows the absorption and luminescence spectra of YAB:Mn at low temperature (*T* = 5 K) in the region of the *R* lines. The spectra have the form of narrow zero-phonon lines (ZPLs) and broad adjacent bands of electron–phonon (vibronic) transitions. Figure 7a demonstrates the evolution of the *R* absorption lines with temperature. The wavelengths of the *R*_1_ and *R*_2_ lines at room temperature—684 nm and 682 nm, respectively—coincide with those reported for YAB:Cr^3+^ [38,39]. Figure 7b shows very weak lines of a spin-forbidden transition from the ground state ^4^*A*_2_ to the next excited (after the ^2^*E* doublet) level ^2^*T*_1_ in the absorption spectrum of YAB:Mn at 5 K. The excitation spectra of the *R* lines are presented in Figure 8. All these experimental data allowed us to determine the energies of the ^2^*E*, ^2^*T*_1_, ^4^*T*_2_, and ^4^*T*_1_ levels; they are provided in Table 3. The values in Table 3, within the precision of measurements, coincide with those reported for Cr^3+^ in YAB [38,39,40]. It is a well-known empirical fact that the strength of the crystal field as well as covalency increases with increased ionic charge [41]. For example, Mn^4+^ in corundum Al_2_O_3_ demonstrates blue shifts of 364, 413, and 2300 cm^−1^ for the *R*_1_, *R*_2_, and *A*_1_–^4^*T*_2_ transitions, respectively, as compared to Cr^3+^ in Al_2_O_3_ (ruby) [41,42]. Both ions substitute for Al^3+^. We tried to find the *R* lines of Mn^4+^ in the spectra of YAB:Mn but failed. It is worth noting that Mn^4+^ in Al_2_O_3_ was introduced together with charge-compensating Mg^2+^.

A broad line at the low-frequency side of the *R*_1_ and *R*_2_ lines of the uncontrolled Cr^3+^ impurity (denoted “*N*” in Figure 7a) noticeably narrows with decreasing temperature and, at low temperatures (*T* < 100 K), it exceeds in amplitude the *R*_1_ and *R*_2_ lines. At the temperature *T* = 5 K, its frequency is 14,571 cm ^−1^. The *N* line apparently refers to a transition in exchange-coupled Cr^3+^-containing pairs. A very similar pattern was observed, for example, in the luminescence spectra of isostructural GdAl_3_(BO_3_)_4_ crystals doped with 1% Cr^3+^ (GAB:Cr^3+^) [17]. The authors attribute the corresponding transition to the emission from the ^2^*E* state of the Cr^3+^-Cr^3+^ pairs. In our case, the formation of Cr-Mn pairs could also be possible.

The inset of Figure 7a shows the absorption spectra at the lowest measured temperature (*T* = 5 K) for two directions of incident light polarization, E||*c* and E⊥*c*. The ratio of the amplitudes of the *R*_1_ and *R*_2_ lines is in agreement with the corresponding ratio for YAB:Cr^3+^ [38] (namely, I(*R*_1_)/I(*R*_2_) = 1 for E||*c*, I(*R*_1_)/I(*R*_2_) = 2 for E⊥*c*), which once again confirms the origin of the observed *R* lines as stemming from the uncontrolled Cr^3+^ impurity. It is also worth noting that we found the same *R* lines of approximately the same intensity in “pure” YAB crystals grown from the same chemicals in the same laboratory as the YAB:Mn crystals under study. The rest of the spectrum observed for YAB:Mn is absent in YAB, so it is obviously associated with manganese.

EPR measurements revealed Mn^2+^ ions occupying yttrium-ion sites in YAB:Mn [28]. Although Mn^3+^ was introduced into the melt solution in the form of Mn_2_O_3_, it must be kept in mind that Mn_2_O_3_ decomposes in air at *T* > 800 °C, losing part of the oxygen—6(Mn^3+^)_2_O_3_ = 4Mn^2+^(Mn^3+^)_2_O_4_ + O_2_—so that Mn^2+^ ions appear. The charge-compensation can be realized by uncontrolled impurities such as Ti^4+^ (see Table 1). Optical spectra of Mn^2+^ in oxide crystals consist, as a rule, of a single broad band corresponding to the ^4^*T*_1_ → ^6^*A*_1_ transition, which for Mn^2+^ in the Y^3+^ position is in the green region of the spectrum [43]. We attribute a broad band peaking at 531 nm (*T* = 10 K, see Figure 5) to the ^4^*T*_1_ → ^6^*A*_1_ transition of Mn^2+^ in YAB:Mn.

Mn^3+^ was not found in the EPR studies of YAB:Mn [28]. Note, however, that Mn^3+^ is a non-Kramers ion and can be studied in some cases only by a special high-frequency EPR technique. Such studies on SrTiO_3_:Mn have shown that Mn^3+^ substitutes for the octahedrally coordinated Ti^4+^ and forms three distinct types of Jahn–Teller centers that differ by charge-compensation mode [44]. The Mn^3+^ ion in octahedral coordination replacing Al^3+^ was found in Al_2_O_3_ (corundum) [45] and Y_3_Al_5_O_12_ (YAG) [20,21]. Below, we discuss the features observed in our spectra of the YAl_3_(BO_3_)_4_:Mn crystal, which we attribute to the transitions in the octahedrally coordinated Mn^3+^ at the Al^3+^ site.

Low-temperature luminescence of YAB:Mn in the IR range (9500–6500 cm ^−1^ or 1055–1500 nm, see Figure 5) consists of relatively narrow (<10 cm^−1^) ZPLs at 9371, 9388, 9430, and 9435 cm^−1^ and an adjacent vibronic band. In addition, narrow lines of uncontrolled impurities of Nd and Sm ions known from the YAB:Nd [3] and YAB:Sm [46] spectra are observed in the spectrum. A similar spectral pattern with narrow ZPLs with frequencies of about 9400 cm^−1^ and a phonon sideband was observed in a number of Mn^3+^-doped garnets and was associated by the authors with ^1^*T*_2_ → ^3^*T*_1_ transitions between excited triplets [20,21,22]. According to the Tanabe–Sugano diagrams [47], levels ^1^*T_2_* and ^3^*T*_1_ have the same dependence on the crystal field, so the energy position of the corresponding transition band is practically independent of the strength of the crystal field. The multiple ZPLs observed in this region of the spectrum are most likely due to both the spin-orbit splitting of the ^3^*T*_1_ level and the orbital splitting of excited triplets caused by the low-symmetry component of the crystal field.

One more relatively narrow (~80 cm^−1^) line associated with manganese is observed in the red part of the low-temperature spectrum at 13,160 cm^−1^ (759 nm) (see Figure 5). It is accompanied by a Stokes vibronic sideband which grows in intensity with rising temperature; simultaneously, an anti-Stokes part appears (see, e.g., [48]). We tentatively assign this line to a transition from the excited orbital triplet ^1^*T*_2_ to the ground Jahn–Teller-split doublet ^5^*E^′^*, ^5^*E^″^* in Mn^3+^ [23]. A similar transition (though not as rich in structure) with a peak at 13,700 cm^−1^ was observed in the low-temperature emission spectrum of Y_3_Al_5_O_12_ (YAG) doped with Mn^3+^ [20]. The excitation spectrum of the PL line 759 nm is presented in Figure 8c. It shows four bands peaking at 17,450, 22,000, 26,750, and 34,326 cm^−1^. Bands at 17,450 and 22,000 cm^−1^ can be related to the spin-allowed transition from the ^5^*E* ground state to the excited ^5^*T*_2_ triplet of Mn^3+^, split by the low-symmetry crystal field, whereas the bands at 26,750 and 34,326 cm^−1^ are apparently associated with the Mn^3+^ transitions to the higher-lying states (^3^*E*, ^3^*T*_1_) [23].

The strongest PL band of Mn^3+^-doped crystals has the maximum in the region of wavelengths 620–670 nm [20,21,22,23] and is associated with the spin-allowed transition ^5^*T*_2_ → ^5^*E.* We assign a broad strong emission band peaked at 15,853 cm^−1^ (631 nm) to the ^5^*T*_2_ → ^5^*E* transition of Mn^3+^. Taking into account positions of the corresponding PLE bands, we find the mean value of 17,725 cm^−1^ as the energy of the ^5^*T*_2_ state.

Based on the experimental values 17,725 cm^−1^ (^5^*T*_2_) and 13,160 cm^−1^ (^1^*T*_2_), as well as the Tanabe–Sugano diagram for the d^4^ configuration [47], we estimate the crystal-field parameter *Dq* and Racah parameter *B* for Mn^3+^ in YAB:Mn as *Dq* = 1785 cm^−1^ and *B* = 800 cm^−1^. The energy difference of ~9400 cm^−1^ between the ^1^*T*_2_ and ^3^*T*_1_ triplets, found from the IR spectra of the ^1^*T*_2_ → ^3^*T*_1_ transition, agrees with these estimates in the framework of the Tanabe–Sugano diagram, which provides additional verification. The value *Dq/B* = 2.23 is very close to *Dq/B* = 2.25 found for Mn^3+^ in garnet-type Ca_3_Ga_2_Ge_3_O_12_ single crystals [22].

## 4. Conclusions

Using XANES and high-resolution optical spectroscopy, the valence composition of Mn ions in YAB:Mn was determined. According to the EXAFS data, manganese is contained in the crystal mainly in the divalent state Mn^2+^(d^5^), and substitutes for Y^3+^. This conclusion is in agreement with the EPR results [28]. Luminescence of the Mn^2+^ ions at the ^4^*T*_1_ → ^6^*A*_1_ transition (near 630 nm) was detected. For charge-compensation reasons, it would be natural to assume that Mn^4+^ is present in a neighborhood of Mn^2+^ [27,49]. It was previously shown for a number of aluminates that Mn^4+^ replaces octahedrally coordinated Al^3+^ [41,49], which is consistent with the proximity of their ionic radii (0.535 Å for Al^3+^ and 0.53 Å for Mn^4+^ [37]). We show that the *R* lines characteristic of the d^3^ configuration (Mn^4+^, Cr^3+^), observed both in the absorption spectra (^4^*A*_1_ → ^2^*E*) and in the luminescence spectra (^2^*E* → ^4^*A*_1_) of YAB:Mn, arise not from Mn^4+^ but from the uncontrolled Cr^3+^ impurity. We failed to find the spectra of Mn^4+^.

During crystal growth, Mn^3+^ was introduced in the form of Mn_2_O_3_, so the presence of the Mn^3+^ ions could be anticipated. In the IR range of the luminescence spectra of YAB:Mn at low temperatures, the spin-forbidden transitions ^1^*T*_2_ → ^3^*T*_1_ and ^1^*T*_2_ → ^5^*E^′^*, ^5^*E^″^* of Mn^3+^(d^4^) were observed. A broad emission band in the orange spectral range (near 630 nm) is associated with the spin-allowed ^5^*T*_2_ → ^5^*E* transition of Mn^3+^. Using the experimental spectroscopic data and the Tanabe–Sugano diagram for the d^4^ configuration, we estimated the crystal-field parameter *Dq* and Racah parameter *B* for Mn^3+^ in YAB:Mn.

Further studies are needed to evaluate the application potential of YAB singly doped with manganese or co-doped with chromium. Our work can serve as a basis for these studies.

## Figures and Tables

**Table 1:** Composition of YAB:Mn determined by X-ray fluorescence analysis. AN—atomic number.

Element	AN	wt.%	Normal. wt.%	Normal. at. %	Error in wt.% (1 Sigma)
Aluminum	13	24.43	21.667	48.17	0.225
Potassium	19	0.08	0.084	0.13	0.006
Calcium	20	0.09	0.090	0.14	0.002
Titanium	22	0.05	0.057	0.07	0.001
Manganese	25	0.75	0.800	0.87	0.009
Iron	26	0.16	0.172	0.18	0.001
Yttrium	39	68.84	73.002	49.25	0.037
Bismuth	83	3.89	4.128	1.18	0.006
		94.3	100	100	

**Table 2:** Parameters of the nearest environment of Mn obtained from EXAFS data.

Model	*R*_f_, %	*S*_0_^2^	Δ*E*_0_, eV	Path	*N*	*R*, Å	*σ*^2^, 10^−3^ Å^2^
Mn in Y position	1.7	0.57 ± 0.18	4.4 ± 2.3	Mn-O	6	2.26(3)	6 ± 5
				Mn-B	6	3.0(1)	10 ± 15
				Mn-O	6	3.16(7)	6 ± 5
				Mn-O	6	3.65(8)	6 ± 5
				Mn-Al	6	3.68(4)	3 ± 6
				Mn-O	6	4.24(8)	6 ± 5
Mn in Y position Mn in Al position	1.6	0.56 ± 0.17	2.8 ± 4.1	Mn-O2	0.7 ± 1.3	2.052	4 ± 7
				Mn-O1	5.3 ± 1.3	2.25(3)	4 ± 7
				Mn-B	6	3.0(1)	4 ± 17
				Mn-O	6	3.1(1)	4 ± 7
				Mn-O	6	3.6(1)	4 ± 7
				Mn-Al	6	3.66(5)	1 ± 6
				Mn-O	6	4.23(9)	4 ± 7

**Table 3:** Energy values (cm^−1^) of the ^2^*E*, ^2^*T*_1_ (at 5 K), ^4^*T*_2_, and ^4^*T*_1_ (at 77 K) levels of uncontrolled Cr^3+^ in YAB:Mn determined from the absorption and excitation spectra.

Level	Cr^3+^ in YAB:Mn
^2^ *E*	14,633
	14,690
^2^ *T*_1_	15,267
	15,312
	15,396
^4^ *T*_2_	16,730
^4^ *T*_1_	23,320

## Data Availability

Data can be obtained from the corresponding author upon reasonable request.
